# Longitudinal Relations Between Representational and Advanced Theory of Mind and Scientific Reasoning From Ages 4 to 7

**DOI:** 10.1111/desc.70270

**Published:** 2026-08-25

**Authors:** David M. Sobel, Özgün Köksal, Christopher Osterhaus, April Moeller Bachhuber, Beate Sodian

**Affiliations:** ^1^ Department of Cognitive and Psychological Sciences Brown University Providence Rhode Island USA; ^2^ Department of Psychology Ludwig Maximilian University of Munich Munich Bavaria Germany; ^3^ Department of Psychology University of Vechta Vechta Lower Saxony Germany

**Keywords:** longitudinal design, scientific reasoning, theory of mind

## Abstract

Children's scientific reasoning involves integrating early‐developing causal reasoning capacities with the metacognitive capacity to represent and update the likelihood of hypotheses given evidence. Numerous studies have shown relations between children's scientific reasoning and certain metacognitive capacities, particularly 6‐ to 10‐year‐olds’ developing theory of mind. Several recent studies suggest, however, that nascent scientific reasoning capacities emerge during the preschool years. The present study examined relations between children's developing representational and advanced theory of mind with their scientific reasoning using a longitudinal design. At 4‐, 5.5‐, and 7.5‐years‐old children received age‐appropriate measures of scientific reasoning, theory of mind, executive function, and language ability. Performance on the theory of mind measures at each age related to children's scientific reasoning. Performance on representational theory of mind measures at 4‐years‐old also predicted children's scientific reasoning later in development, both directly at 5.5‐years‐old and indirectly through performance on more advanced theory of mind measures at 7.5‐years‐old. These relations held controlling for the measures of executive function and language ability, which also related to children's scientific reasoning. These findings point to the onset of representational theory of mind as being vital to the origins of children's scientific reasoning. As children's representational capacities develop during the preschool and early elementary‐school years, children's scientific reasoning capacities become more advanced.

## Summary

- A longitudinal study found relations of theory of mind and scientific reasoning at the ages of 4, 5.5. and 7.5 years.
- At age 4, a battery of representational (first order) theory of mind (RToM) measures related to children's scientific reasoning at that age.
- At ages 5.5 and 7.5 Advanced (AToM) measures related to children's concurrent scientific reasoning.
- Longitudinally, RToM at age 4 related to children's scientific reasoning at age 5.5, independent of the emergence of AToM at age 5.5.
- RToM at age 4 did not directly relate to scientific reasoning at age 7.5; it appeared to be mediated by AToM performance at that age.
- Executive function showed independent concurrent relations with scientific reasoning at ages 4 and 5.5, as well as a longitudinal relation from age 5.5 to scientific reasoning at age 7.5.

## 1 Introduction

Scientific reasoning refers to the processes involved in generating, testing, and evaluating explanations about the world through evidence‐based inquiry. It encompasses forming hypotheses, designing experiments to discern among hypotheses, interpreting data from experiments, and potentially revising beliefs based on interpretations (e.g., Schauble et al. 1995). Numerous researchers have suggested that children's capacity for scientific reasoning emerges from integrating their causal inference abilities with their developing capacities for mental state representation and reasoning, particularly their ability to reflect on, interpret, and change their own beliefs (e.g., Booth et al. 2022; Koerber et al. 2015; Kuhn 2010; Shavlik et al. 2022; Shtulman and Walker 2020; Sodian et al. 2018; Weisberg and Sobel 2022).

Children's causal knowledge and causal reasoning are thought to be both sophisticated and early emerging during infancy (see e.g., Basch and Wang 2024; Goddu and Gopnik 2024, for reviews). By contrast, the capacities for mental state representation that are required for scientific reasoning, referred to as *Advanced Theory of Mind* (Osterhaus and Bosacki 2022; Osterhaus and Koerber 2021) typically develop after the preschool years. These capacities include children's ability to interpret data actively, including their broader epistemic understanding and interpretation of ambiguous events (e.g., Carpendale and Chandler 1996; Heiphetz et al. 2013; Kuhn et al. 2000; Mitroff et al. 2006) as well as children's understanding that belief states are recursive and can themselves be about beliefs (Perner 1991; Perner and Wimmer 1985).^1^

To illustrate these relations, consider a prototypical example of children's scientific reasoning—their use of the “control of variables strategy” (henceforth, *CVS*). *CVS* involves children generating and testing hypotheses about causal mechanisms while also reflecting on the validity of their predictions and intervention. Second graders (i.e., 8‐year‐olds) can use *CVS* if they have been explicitly taught the strategy (Chen and Klahr 1999), and choose unconfounded experiments to learn causal relations (e.g., Bullock and Ziegler 1999). Younger children tend to find measures that test their understanding of *CVS* challenging (e.g., Croker and Buchanan 2011; Tschirgi 1980; Zimmerman 2007). Elementary‐school‐aged children do not necessarily use this strategy spontaneously when trying to learn a causal system (e.g., Klahr et al. 2011; Kuhn et al. 2008; Kuhn and Dean 2005; Schauble 1990; see Schwichow et al. 2016, for a review).

There are well‐established relations between elementary‐school‐aged children's AToM and their ability to use CVS. Osterhaus et al. (2017) found that AToM scores in 2nd‐ and 4th‐graders related to their experimentation skills, including using *CVS*.^2^ Koerber and Osterhaus (2019) found performance on AToM measures related to 6‐year‐olds’ use of *CVS*, independently of general information processing skills (see also Lecce et al. (2025) for similar findings on 8–10‐year‐olds). In longitudinal work, Osterhaus and Koerber (2023) found that 6‐year‐olds (but not 5‐year‐olds), who were competent on second‐order false belief measures, were better at using *CVS* than children who did not succeed on such false belief measures; these children's AToM scores at Age 6 also predicted their scientific reasoning skills at Ages 9–10.

More recently, however, researchers have suggested that the ability to use *CVS* emerges and develops between the ages of 4–7. For example, van der Graaf et al. (2015) asked 4‐ to 6‐year‐olds to design experiments using a simplified, scaffolded version of the Ramps task used by Chen and Klahr (1999). Children were asked to determine which of four potential causes was efficacious by designing interventions and receiving feedback. Roughly half of the 5‐ and 6‐year‐olds could design unconfounded experiments. The younger children they investigated struggled to do so, suggesting that development does occur, just earlier than expected (see also Sobel et al. 2022; van der Graaf et al. 2016, 2018, for similar findings). Between the ages of 3–6, children develop the capacity to choose to observe the results of an unconfounded intervention over a confounded one when asked to learn about a causal relation (e.g., Lapidow and Walker 2020; Moeller et al. 2022; Shavlik et al. 2022). Moeller et al. (2022) also found that young children's ability to recognize that evidence they had observed was unconfounded regarding a particular causal conclusion was unrelated to their ability to choose what intervention would produce unconfounded data. In these cases, children were presented with a set of possible hypotheses, and asked to infer what data they would need to see in order to disambiguate among them.

Other facets of scientific reasoning are also developing during this time. Between the ages of 4–6, children begin to understand that hypotheses can be tested (e.g., Köksal and Sodian 2023; Piekny et al. 2014; Sodian et al. 1991). Five‐ and 6‐year‐olds recognize when they observe confounded evidence, they lack sufficient information to make appropriate causal conclusions about those data (Köksal et al. 2021). At these ages, children also recognize that data can be manipulated to lead to false conclusions (e.g., Koerber et al. 2005; Ruffman et al. 1993), and that hypothesis‐testing involves understanding what kind of data one needs to observe (Morris et al. 2012). By Age 4, children refute causal claims that are inconsistent with observed data (Köksal‐Tuncer and Sodian 2018). Thus, before children start formal schooling, they understand what data are needed to make causal conclusions given a set of hypotheses, which indicates a nascent scientific reasoning ability.

### 1.1 The Role of Theory of Mind in the Development of Scientific Reasoning

AToM capacities, which have been established to relate to children's scientific reasoning in elementary school, build on preschoolers’ earlier‐developing *Representational Theory of Mind* (*RToM)*. RToM involves children's ability to appreciate that they themselves and others can have false beliefs about the world (e.g., Perner 1991; Wellman 1990) and their transition from appreciating the mind as passively observing information to one that actively interprets events (e.g., Flavell et al. 1995; Sodian and Wimmer 1987). RToM emerges when children are approximately 4‐years‐old^3^ (Wellman et al. 2001), and RToM and AToM are directly related developmentally (e.g., Osterhaus et al., 2026; Osterhaus and Koerber 2021). The importance of RToM, however, has rarely been investigated with respect to children's emerging scientific reasoning. The question that motivates the present study is whether foundational RToM abilities are important individually and in conjunction with AToM skills for the development of scientific reasoning abilities.

Why might RToM relate to children's emerging scientific reasoning ability? RToM provides children with the understanding that beliefs can be false, that false beliefs originate from mistaken or insufficient information, and that beliefs, rather than states of reality, are causal determinants of agents’ actions (e.g., Perner 1991; Wellman and Liu 2004). The emergence of RToM is also linked to understanding the relation between two (or more) different representations of the same object or entity more generally, as in dual labelling, visual perspective taking, interpreting ambiguous figures, and overcoming mutual exclusivity—all capacities that develop around Age 4 in conjunction with success on false belief measures (see e.g., Doherty and Perner 2020).

Scientific reasoning involves understanding that representations of states of the world (i.e., hypotheses or the potential causal relations among events) may be wrong, and that alternative representations of cause‐effect relations are possible and may be evaluated based on observing data. The representational capacities indicated by success on RToM measures allow children to hold these multiple hypotheses in mind to be evaluated. If children can only hold one hypothesis in mind at the time (and if they cannot distinguish a hypothesis from reality), then they would be unable to understand the relation between hypotheses and evidence. RToM, however, seems insufficient for constructing and representing a set of possible hypotheses that serve as potential explanations of the data and then reflecting on whether each hypothesis is consistent with current data under consideration. This capacity further requires understanding that hypotheses have epistemic value, originate from one's perceptions or inferences, and generate expectations about events in the world, which require explanation or updating when violated. Forming and evaluating a set of possible hypotheses requires the ability to represent a set of beliefs recursively, assign them distinct epistemic values based on prior knowledge, and consider different possible interpretations of evidence‐that is, AToM. We would not expect direct relations between RToM and all kinds of scientific reasoning. Instead, RToM potentially relates to determining what evidence is needed to evaluate hypotheses when the hypotheses are presented to the child.

### 1.2 The Present Study

The present study examines the extent to which children's RToM and AToM relate to their emerging capacities for scientific reasoning during the preschool years. We tested children longitudinally at Ages 4, 5.5, and 7.5. At Age 4, children received a set of RToM measures as well as a strategy‐choice scientific reasoning measure that considered whether they understood that data were confounded (Moeller et al. 2022). At Age 5.5, they received the RToM measures and one AToM measure; they also received the same scientific reasoning measure as at Age 4, as well as the (supported) strategy‐production version of the Ramps task used by van der Graaf et al. (2015). Finally, at 7.5‐years‐old, they received the same Ramps task, but also a set of contextualized *CVS* questions in an inventory of scientific reasoning (Koerber and Osterhaus 2019), as well as a battery of AToM measures. At each age, children also received a set of age‐appropriate measures of executive function, and at 4‐years‐old and 7.5‐years‐old, children received standardized measures of language ability. Executive function has long been thought to correlate with theory of mind development (e.g., Carlson and Moses 2001; Hughes and Ensor 2007), as has language ability (e.g., Astington and Jenkins 1999; de Villiers and de Villiers 2014). Finally, when children were 5.5‐ and 7.5‐years‐old, they were given a measure of scientific content knowledge. van der Graaf et al. (2015) found that children's ability to evaluate evidence at Age 5 predicted their scientific content knowledge at Age 6. In contrast, Koerber and Osterhaus (2020) found that science content knowledge at Age 5 predicted scientific reasoning at Age 6, but not the reverse.

This design allows us to consider several research questions. First, we examine whether there is a relation between RToM and scientific reasoning in children as young as 4‐years‐old. Doherty and Perner (2020) and Perner (2025) argue that RToM capacities indicate the ability to hold multiple, distinct representations. This description seems to parallel the ability to represent multiple hypotheses in scientific reasoning.

Second, we examine whether relations between RToM and scientific reasoning are present longitudinally after Age 4. Given, however, that there are links between RToM development and the emergence of AToM (e.g., Devine et al. 2016; Lecce et al. 2014; Peterson and Wellman 2019), and given that AToM capacities relate to children's scientific reasoning, it is possible that any direct relation between RToM at Age 4 and later‐developing scientific reasoning capacities (at Age 5.5 or 7.5) is mediated by the emergence of AToM. Our design allows us to consider these relations.

Third, we investigate whether executive function has similar relations to scientific reasoning at each age group, as well as whether early‐emerging executive function relates longitudinally to later‐developing scientific reasoning or is mediated by concurrent relations with executive function, and whether executive function also mediates any longitudinal relations between theory of mind and scientific reasoning. Finally, although not the primary focus of our investigation, including measures of scientific content in our analysis sheds light on its influence on children's developing scientific reasoning.

## 2 Methods

### 2.1 Participants

The present study was part of a larger longitudinal project examining children between the ages of 4 and 7.5. The initial sample at the first measurement point (T1) comprised two hundred three 4‐year‐olds (*M* = 48.00 months, *SD* = 1.53, range = 44–51, 90 girls, 113 boys). One hundred eighty 5.5‐year‐olds participated in the second measurement point (T2; *M* = 64.97, *SD* = 1.45, range = 61–70, 82 girls, 98 boys). One hundred fifty‐eight 7‐year‐olds participated in the third and final measurement point (T3; *M* = 88.93, *SD* = 1.52, range = 86–96, 73 girls, 86 boys).

All children were tested in a major metropolitan area in Germany and all interviews were conducted in German. An additional 19 children were initially recruited. Seventeen children were excluded due to insufficient German language skills assessed by a standardized language test (Grimm et al. 2015). Two additional children refused to take part in the study. Children were recruited from predominantly White, middle‐class families through local birth registries. No child had any diagnosed developmental disorders or hearing impairments. Other information about the sample is provided in the Supporting Information.

### 2.2 Materials and Procedures

**FIGURE 1:**
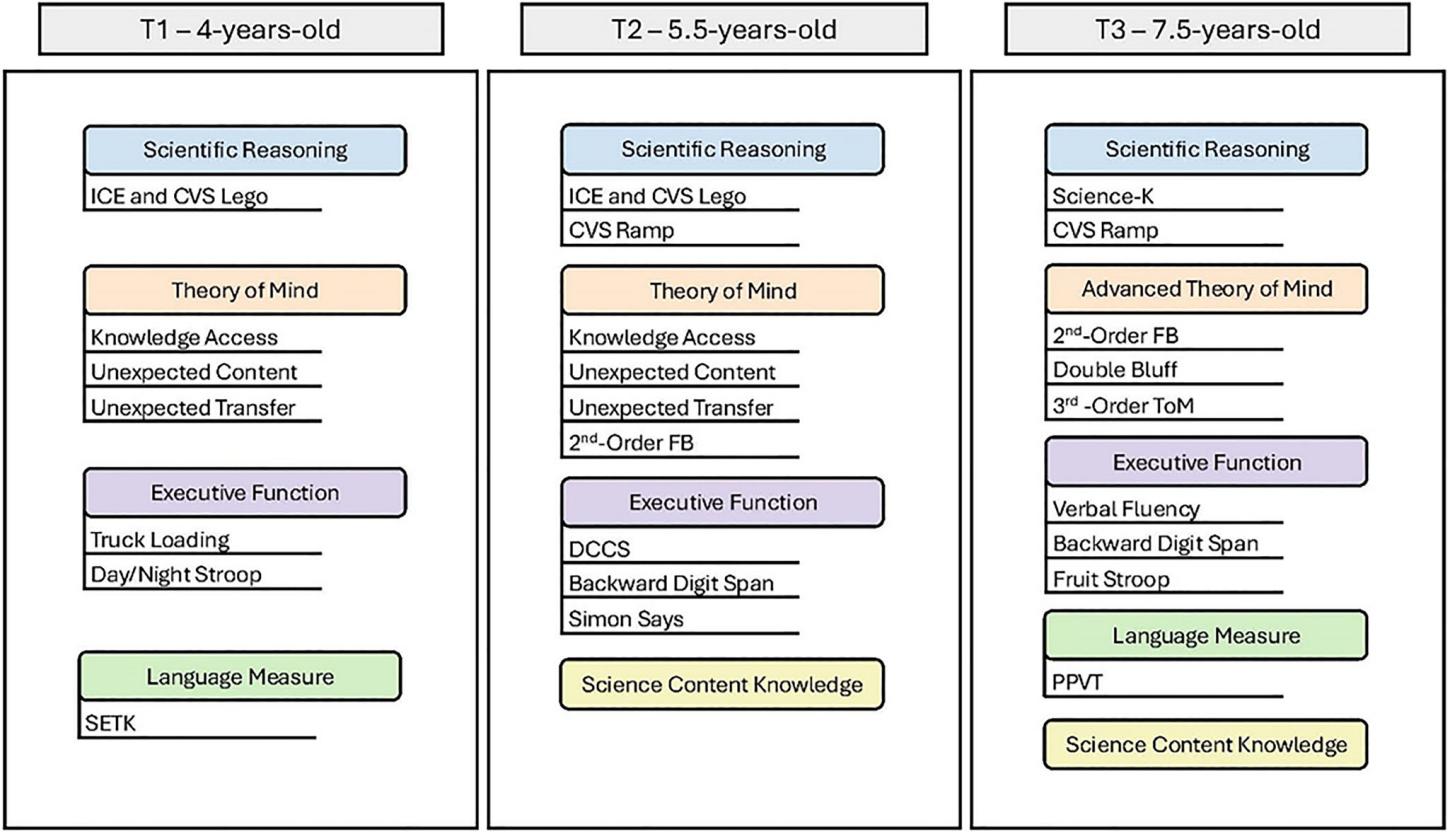
Overview of the methods at each time point.

The study received approval from the ethics committee of the [Faculty of Psychology and Education, Ludwig‐Maxmilian University of Munich, Germany]. Parents provided consent through signed forms, and children provided verbal assent following the university ethics committee guidelines. Each child received a personal gift for their participation at each measurement point. Parents were compensated for their travel expenses. An overview of the tasks children received is shown in Figure 1. This study was not preregistered and thus all analyses should be considered exploratory.

#### 2.2.1 Measures at the First Time Point (T1, Approximately 4‐Years‐Old)

##### 2.2.1.1 Scientific Reasoning Measure

We assessed children's scientific reasoning with tasks from Moeller et al. (2022): an Interpretation of Confounded Evidence (ICE) task, and a 2‐ and 3‐variable Control of Variables task (*CVS2/CVS3*). Children interacted with Lego Duplo bricks and a custom‐built light‐up box that activated when objects were placed on it, controlled by the experimenter. Children were introduced to the lightbox and shown that some bricks made the box light up (“lighters”) and some did not (“non‐lighters”). They were also shown that combinations of bricks made the box light up as long as one of those bricks was a lighter.

##### 2.2.1.2 Interpretation of Confounded Evidence Task

The experimenter placed a stick of four bricks on the box horizontally, so all bricks touched the box, which activated. This stick, as well as all sticks used in the procedure, were glued together so that the bricks could not be separated. Children were asked if they knew which of the bricks in this stick made the box light up, whether they were certain, and to provide an explanation for their answer. The more appropriate answer is “no” because even though the machine activates for the stick, these data do not allow children to recognize whether any individual brick is efficacious. Scoring the explanation allowed us to measure whether children understood the ambiguity of the evidence they observed. On each trial, children received a point if they responded in the more appropriate manner, and another point if they justified their response by stating that they could not know which brick(s) made the box activate because the individual efficacy of each brick could not be determined individually.

##### 2.2.1.3 Control of Variables Tasks

In the two‐variable task, the experimenter placed a stick of two bricks horizontally on the box, which activated. The experimenter pointed to the top brick and said, “We want to find out if this brick makes the box light up.” The stick was placed in front of the child. Two additional sticks were then placed on the table. One (the more appropriate choice) swapped the top brick with a novel color but kept the bottom brick the same. The other (the less appropriate choice) swapped both bricks, resulting in a stick of two novel colors. The experimenter presented the choices and said, “You can pick one of these sticks to place on the box to find out if the top brick makes the box light up. Which stick is best to find out if the top brick makes the box light up?” At this point, children were not shown any data, so the more appropriate response involves them reasoning about what possible data they could observe. The more appropriate choice has the potential to be informative about the target brick, while the less appropriate choice provides no information. After children indicated their choice, the experimenter asked, “Why is this the best stick to put on the box?” Children received a point if they made the more appropriate choice, and another point if they justified their choice in terms of the absence of the test brick in the choice, the presence of the control brick(s) in the choice, or both (indicating that they understood the difference between what they had seen efficacious and what they were now about to test). The experimenter then placed the chosen stick horizontally on the box, and the box did not light up, thus allowing children to see evidence for the trial.

**FIGURE 2:**
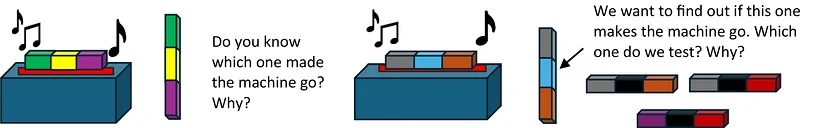
Examples of interpretation of confounded evidence (left) and control of variables trials (right) in Lego task used at Time 1 and Time 2.

The procedure for the three‐variable task was the same as for the two‐variable task, except that children were shown that a stick of three bricks made the box light up, were asked to find out if the middle brick made the box light up, and were given three sticks as choices. The more appropriate choice again varied the brick in question and kept the other two bricks the same; a second stick varied the brick in question as well as an additional brick; the third stick varied all three bricks, resulting in a stick with three novel colors. A fixed task order was used, such that children always received the tasks in the following order: ICE–CVS2–CVS3–CVS2–CVS3–ICE (examples of each trial type shown in Figure 2). The scores on these six trials were summed to create a single measure of scientific reasoning, ranging from 0 to 12.

##### 2.2.1.4 Theory of Mind Measures

We assessed children's theory of mind reasoning at T1 with three tasks taken from the German Theory of Mind Scales measure (Hofer and Aschersleben 2007): knowledge access, content false belief (i.e., unexpected contents), and location false belief (i.e., unexpected transfer). Children also received two executive function measures and a set of measures of general language comprehension and cognition (the SETK). Detailed instructions for all of these tasks are given in the Supporting Information section.

#### 2.2.2 Measures at the Second Time Point (T2, Approximately 5.5‐Years‐Old)

##### 2.2.2.1 Scientific Reasoning Measure

**FIGURE 3:**
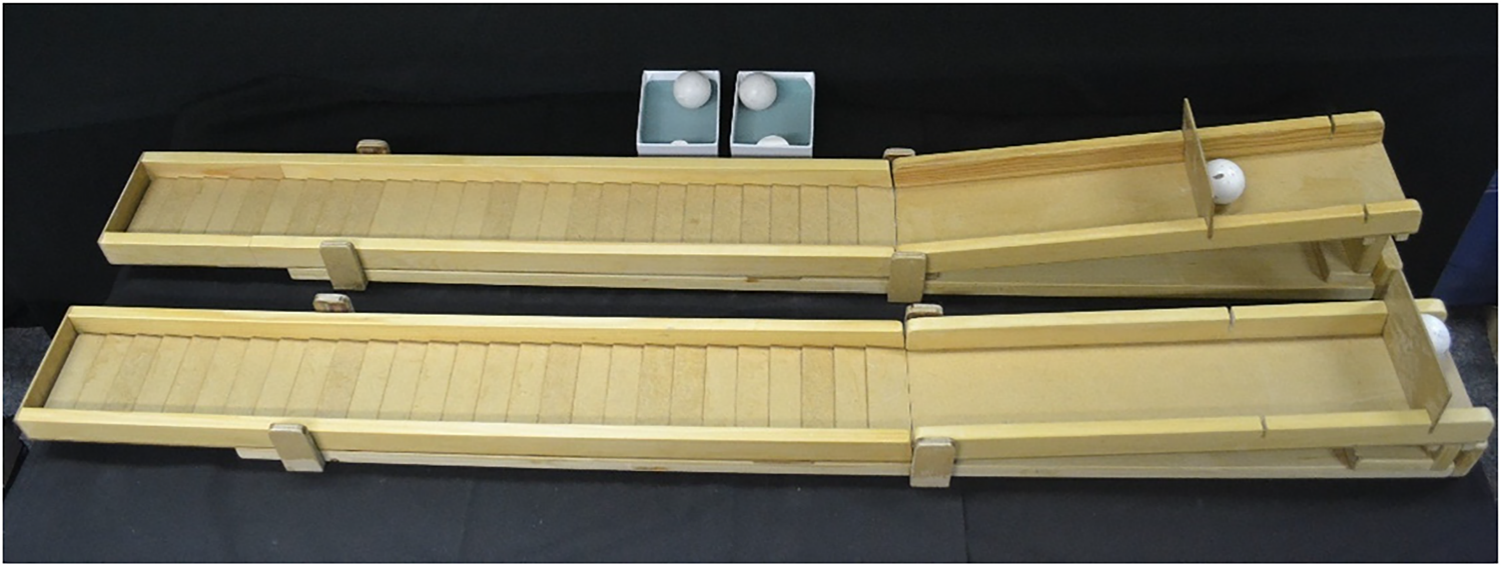
Ramps used in the scientific reasoning measure at Times 2 and 3. Each ramp has three different variables with two possible settings. In the top side of the photo, the ball is light, the slope is steep, and the starting gate is placed further down. In the bottom side of the photo, the ball is heavy, the slope is less steep, and the starting gate is placed near the top.

The same ICE and *CVS* Lego measure from Time 1 was administered to children at this time. We also administered a version of the *Ramps task* adapted from van der Graaf et al. (2015) for testing children's experimentation abilities. Children interacted with two wooded ramps (shown in Figure 3), and were asked to design experiments to test the effect of different variables on the distance balls rolled. Our procedure followed van der Graaf et al. (2015) approach with two modifications: We did not ask children to explain their design decisions and we included three variables (instead of four) to reduce task duration.

The task consisted of three difficulty levels, each involving three trials, resulting in a total of nine trials of experimental design. In each trial, children were asked to find out whether a target variable influenced the distance a ball rolled down the ramp. At the first level, we assessed children's ability to design *contrastive tests* by having them compare two different settings of a target variable. The other two variables were set in the same manner for both ramps. At the second level, we evaluated children's capacity to design *controlled tests with two variables*, where the target variable differed while the other variable remained constant for both ramps. At the third level, we considered how children designed *controlled tests with three variables*, where the target variable should be varied while keeping the other two variables constant for both ramps. If children's initial design was incorrect, they were given a second try. After each trial, the experimenter provided instructional feedback on the correctness of the design.

For each trial, children received a score of 2 if their design was correct on the first try, a score of 1 if it was correct on the second try, and a score of 0 otherwise. The experiment score was calculated based on the sum of nine experiments, resulting in a score ranging from 0 to 18. If all the experiment designs were incorrect at a specific difficulty level, the subsequent levels were not administered, and the children received a score of 0 for the remaining trials. The measure was prematurely terminated for 19 children for this reason, with most occurring during the third difficulty level. These children received partial scores, which we included in our analyses. The interrater reliability between the two coders, intraclass correlation coefficient (ICC), was 0.99.

##### 2.2.2.2 Other Measures

The same RToM measures from T1 were administered at T2, along with a fourth measure of AToM, a second‐order false belief task. Three Executive Function measures were also administered. These methods are described in detail in the Supporting Information section.

Finally, children were given a 17‐item scientific content measure, assessing their knowledge about science concepts such as melting, evaporation, and freezing (based on Steffensky et al. 2012, adapted by Koerber and Osterhaus 2019). All questions were verbally presented to the children, and the answer choices were visually illustrated. Children received a score of 1 for a correct response on each item (Except for one where children could get a partial score [0.5]). As an example, one question asked why ice cubes melt in water. Children were presented with three options: because the water is warmer than ice cubes (scored a 1), because the water is more liquid than ice cubes (0), or because the water is more transparent than ice cubes (0). Because some children had a few missing item scores, we computed a proportion correct score. ICC was 0.98. To provide a preview of the analyses, the scientific content measure did not relate to the theory of mind, executive function, or scientific reasoning measures in the final analysis. These analyses will be presented in the Supporting Information section.

#### 2.2.3 Measures at the Third Time Point (T3, Approximately 7.5‐Years‐Old)

##### 2.2.3.1 Scientific Reasoning Measure

**FIGURE 4:**
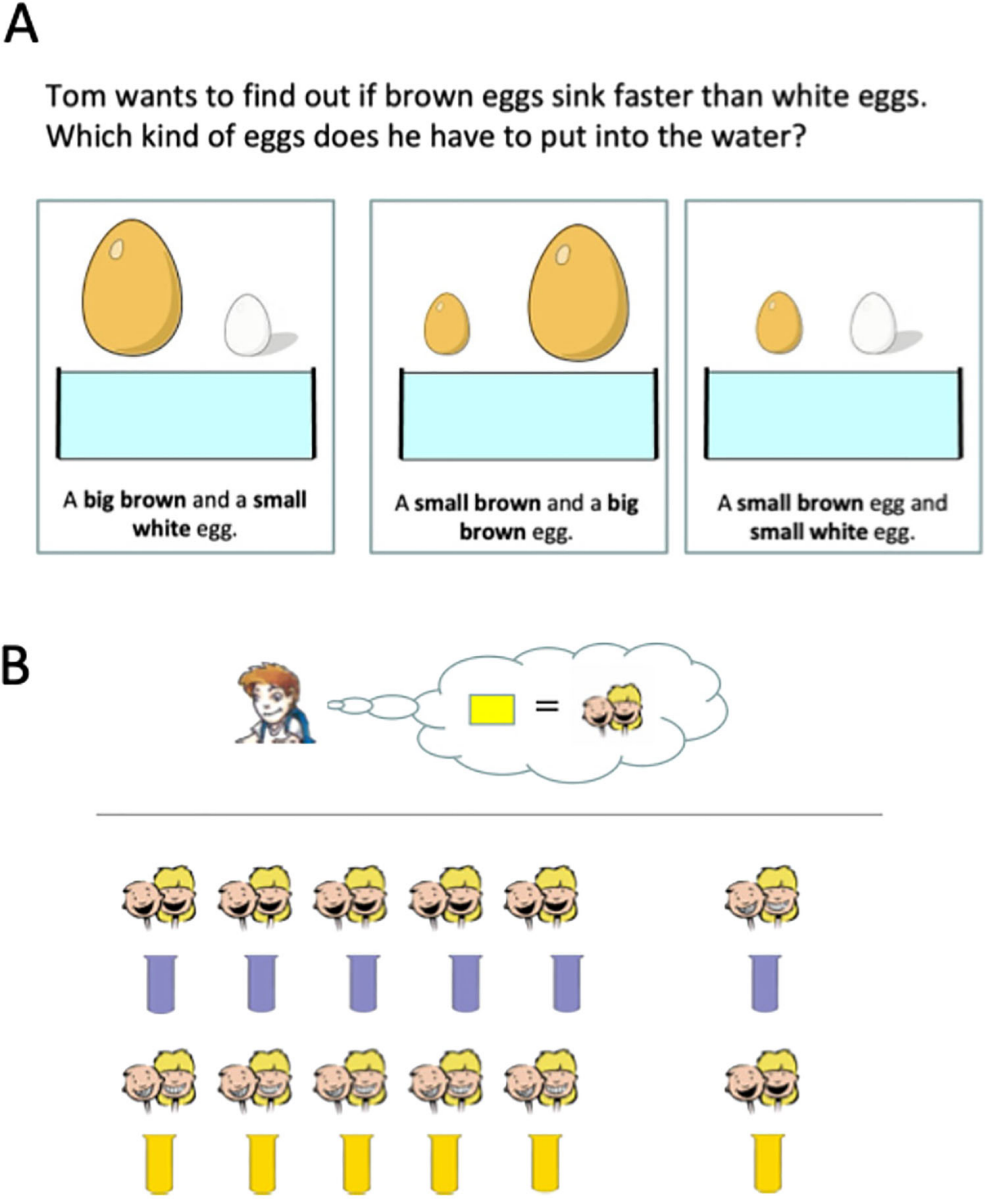
Sample items from the Science‐K Inventory. (A) Sample item assessing experimentation skills using CVS. Children are asked to determine how to test whether brown eggs sink faster than white eggs by choosing between different experimental setups. (B) Sample item assessing data interpretation skills through covariation. Children must assess whether the protagonist's belief that yellow juice causes bad teeth is supported by the data, which actually shows that purple juice is responsible. Reprinted from Osterhaus and Koerber (2023).

The same modified version of the Ramps task was administered and coded as in Time 2. ICC was 0.97. In addition, we administered two subscales of the “*Science‐K*” task (Koerber and Osterhaus 2019; Osterhaus and Koerber 2023); http://www.osf.io/b5mr8, which assessed children's experimentation skills (8 items) and their ability to interpret data and use CVS (7 items). An example experimentation item is shown in Figure 4. The experimenter told the child about a protagonist who sought to “find something out” about a given phenomenon. Upon conclusion of the story, children were asked what the protagonist should do to find out and presented a forced choice among one correct response and two distractors, which either fell short of experimental control or isolated the wrong variable. Afterward, a control question was asked to ensure children's engagement (“Can you tell me again what [protagonist] wanted to find out?”). We scored responses for each item as either incorrect (0) or correct (1), with correct responses also indicating that the child responded correctly on the control question. Interrater reliability was perfect (1.0).

##### 2.2.3.2 Other Measures at T3

We administered a battery of Advanced Theory of Mind measures, Executive Function measures, and the German version of the Peabody Picture Vocabulary Test. All of these are described in detail in the Supporting Information section. Finally, children were given a longer version of the scientific content measure used at Time 2 (18 items, different from those used at Time 2). Interrater reliability was excellent (ICC = 0.98).

## 3 Results

Table 1 shows performance on each measure at each time point. To account for missing data and to integrate the scoring of the measures, we created composite scores for children's theory of mind and executive function performance (detailed in the Supporting Information section). Although the study was not preregistered, the factor analyses reported in the Supporting Information section suggest to use these composite scores to measure the role of theory of mind and executive function at each time point, which is how we conducted the study. We make one deviation at Time 2, which we will highlight below.

**TABLE 1:** Summary of performance on all measures (percentage correct, except where indicated; standard deviations in parentheses).

Measure	Time 1		Time 2		Time 3	
	4 years old		5.5 years old		7.5 years old	
Theory of mind Measures						
	Knowledge	75	Knowledge	94		
	Access	(44)	Access	(23)		
	Contents	38	Contents	66	Double	78
	False belief	(49)	False belief	(48)	Bluff	(41)
	Explicit	42	Explicit	72	3rd Order	76
	False belief	(49)	False belief	(45)	False belief	(43)
			2nd Order	35	2nd Order	62
			False belief	(48)	False belief	(49)
Executive function Measures						
	Day/Night	73	Simon says	52	Fruit stroop	29.94s
	Stroop (Inhibition)	(25)	(Inhibition)	(28)	(in Secs; Inhibition)	(8.85)
	Truck	42	DCCS	61	Verbal	30.28
	Loading	(29)	Border	(17)	Fluency	(7.51)
	(Planning)		(Planning/Set shifting)		(Sum score; Planning)	
			BDS (mean capacity; working memory)	4.20	BDS (mean capacity; working memory)	6.28
				(1.74)		(1.25)
Scientific reasoning Measures						
	Lego task	32	Lego task	47		
		(16)		(21)		
			Ramps task	42	Ramps task	74
				(17)		(20)
					Science K	60
					Overall	(16)
Scientific Content Measures						
			Content	47	Content	62
			Measure	(18)	Measure	(18)
Cognitive/Language Control measures						
	SETK	54.97			PPVT	38.58
	Range 37–73	(7.77)			Range 22–51	(6.54)

### 3.1 Relations Among Variables at Time 1

Theory of mind scores significantly correlated with children's performance on the CVS Lego measure, *r*(172) = 0.25, *p* < 0.001. Theory of mind scores also significantly correlated with children's age, *r*(187) = 0.19, *p* = 0.009, as well as their executive function score, *r*(186) = 0.29, *p* < 0.001 and SETK score, *r*(178) = 0.39, *p* < 0.001. The executive function measures also correlated with the SETK score, *r* (188) = 0.22, *p* = 0.002 and with the CVS measure, *r(*183) = 0.19, *p* = 0.01. Finally, children's gender significantly correlated with performance on the *CVS* measure, with boys performing worse on the CVS measure than girls, *r*(184) = –0.16, *p* = 0.03.

To isolate the relation between theory of mind and children's scientific reasoning at this time point, we constructed a hierarchical regression model. Performance on the CVS Lego measure was the dependent variable; children's age, gender, EF score and SETK score were the independent variables. This model explained a significant amount of variance, *R* ^2^ = 0.09, *F*(4, 162) = 4.01, *p* = 0.004. We then added children's theory of mind score, which significantly explained more variance Δ*R* ^2^ = 0.03, *F*(1, 161) = 4.79, *p* = 0.03. Children's theory of mind related to their scientific reasoning, controlling for these other measures, *β* = 0.19, *t* = 2.19, *p* = 0.03. In this final model, children's executive function score 3.5.1 also explained a significant amount of variance on its own, *β* = 0.17, *t* = 2.15, *p* = 0.03. The SETK score, however, was not significant in this model, *β* = 0.02, *t* = 0.30, *p* = 0.77.

### 3.2 Relations Among Variables at Time 2

There was a significant correlation between children's age and theory of mind score, *r*(178) = 0.17, *p* = 0.02, and age and executive function score, *r*(178) = 0.20, *p* = 0.007. Theory of mind and executive function scores were also significantly correlated, *r*(178) = 0.21, *p* = 0.005. Further, gender was related to their executive function scores, with girls outperforming boys, *r*(178) = –0.17, *p* = 0.02.

Performance on the *CVS* Lego and Ramps tasks was correlated, *r*(182) = 0.23, *p* = 0.003, and these measures were averaged to form a scientific reasoning score. This score was significantly correlated with the executive function measures, *r*(178) = 0.33, *p* < 0.001, and marginally correlated with theory of mind scores, *r*(178) = 0.13, *p* = 0.09. We conducted a regression with children's scientific reasoning score as the dependent variable and their executive function and theory of mind scores as the independent variables. The overall model was significant, *R* ^2^ = 0.12, *F*(2, 177) = 11.50, *p* < 0.001. Only the executive function score was a significant individual predictor, *β* = 0.32, *t* = 4.46, *p* < 0.001.

A difference between the theory of mind measures administered at Time 1 and Time 2 was the presence of the more age‐appropriate AToM measure (the second‐order theory of mind task) at Time 2. As described in the Supporting Information, children's overall theory of mind score at Time 2 might have weighed more heavily on their RToM performance than this AToM measure. To investigate whether the emergence of AToM specifically related to children's scientific reasoning, we separated performance on the RToM measures from the second‐order false belief measure. The former did not significantly correlate with scientific reasoning score, *r*(178) = 0.04, *p* = 0.59, but the latter did, *r*(165) = 0.23, *p* = 0.004. We built similar regression models with scientific reasoning score as the dependent variable and their executive function and this AToM score as the independent variables. Children's executive function score was a significant predictor, *β* = 0.29, *t* = 3.91, *p* < 0.001, as was performance on the second order theory of mind measure, *β* = 0.17, *t* = 2.24, *p* = 0.02. We also separated performance on the *CVS* Lego and *CVS* Ramps tasks and considered these relations individually. Only the Ramps task significantly correlated with the second order theory of mind measure, *r*(163) = 0.22, *p* = 0.005. We conducted a regression with performance on the Ramps task as the dependent variable and performance on the Lego task, second‐order ToM performance, and executive function as independent variables. This analysis showed that only performance on the second‐order ToM measure significantly predicted performance on the Ramps task, *β* = 0.16, *t* = 2.02, *p* = 0.04.

### 3.3 Relations Among Variables at Time 3

We performed a principal components analysis on performance on the Ramps task and Science K batteries at Time 3. These three scores could be reduced to a single factor, so performance on each measure was z‐scored and averaged based on the weight of that factor to the component, thus creating an overall scientific reasoning score at Time 3.

This scientific reasoning score significantly correlated with children's theory of mind score, *r*(156) = 0.36, *p* < 0.001 and PPVT score, *r*(153) = 0.29, *p* < 0.001, but not their executive function, *r*(157) = 0.11, *p* = 0.17 or age, *r*(156) = 0.12, *p* = 0.13. We conducted a regression with children's scientific reasoning score as the dependent variable, and age, theory of mind, PPVT score, and executive function as independent variables. The overall model was significant, *R* ^2^ = 0.17, *F*(4, 148) = 7.54, *p* < 0.001. Theory of mind was a significant predictor, *β* = 0.28, *t* = 3.47, *p* < 0.001 as was PPVT score, *β* = 0.19, *t* = 2.31, *p* = 0.02. Age, *β* = 0.09, *p* = 0.26, and Executive Function, *β* = 0.03, *p* = 0.69, were not significant.

### 3.4 Interim Summary

There were significant relations between scientific reasoning measures administered to children at each age and their theory of mind scores. At 4‐years‐old, there was a significant relation between performance on the CVS Lego task and RToM measures. At 7.5‐years‐old, there was a significant relation between performance on the scientific reasoning measures and AToM measures. At 5.5‐years‐old, the AToM measure, but not the RToM measures significantly correlated with scientific reasoning, and specifically only for the Ramps task.

We interpret these data as suggesting a particular developmental trajectory: RToM at Age 4 relates to reasoning on the Lego task, which involves the understanding that data can be confounded as well as the choice of interventions to determine an unconfounded conclusion. AToM at Age 7.5 predicts more complex scientific reasoning, as measured on the Science K and Ramps task. Critically, this capacity is emerging around Age 5.5, and is related to these emerging AToM capacities.

What we have not done yet is specify the relations between theory of mind and scientific reasoning longitudinally, controlling for children's scientific reasoning at prior ages. We would like to consider relations among the scientific reasoning measures at each time point, and whether earlier theory of mind or executive function abilities relate to children's scientific reasoning capacities at later ages. We turn to these analyses in the next section.

### 3.5 Longitudinal Relations Involving Scientific Reasoning

***3.5.1 Relations Between Time 1 and 2***: We first considered relations between Age 4 and 5.5. Scientific reasoning at Age 5.5 was significantly correlated with performance on the *CVS* Lego task at Age 4, *r*(163) = 0.17, *p* = 0.03, performance on the SETK measure at Age 4, *r*(168) = 0.20, *p* = 0.009, as well as performance on the theory of mind measures, *r*(171) = 0.30, *p* < 0.001 and the executive function measures at Age 4, *r*(177) = 0.20, *p* = 0.006. We conducted a regression with children's scientific reasoning score at Time 2 as the dependent variable, and age at Time 1, gender, performance on the SETK, performance on the executive function measures and the theory of mind measures at both Times 1 and 2, as well as performance on the *CVS* Lego measure at Time 1 as the independent variables. The overall model was significant, *R* ^2^ = 0.17, *F*(8, 143) = 3.77, *p* < 0.001. Only theory of mind at Time 1, *β* = 0.23, *t* = 2.51, *p *= 0.01, and executive function at Time 2, *β* = 0.26, *t* = 3.08, *p = *0.003, were significant predictors. Following the analysis separating RToM and AToM at Time 2 presented above, when only the second order theory of mind measure at Time 2, however, was used instead of the overall ToM score at Time 2 in this model, it was also a unique predictor of scientific reasoning at Time 2, *β* = 0.18, *t* = 2.22, *p = *0.03.

Importantly, scientific reasoning at Time 1 did not relate to scientific reasoning at Time 2. This pattern of results held even when looking at only performance on the Lego task between Time 1 and Time 2. This suggests that the way in which children are engaging in scientific reasoning at Age 4 and Age 5.5 might be fundamentally different, and not directly related to one another. Rather, each might be more related to children's developing theory of mind abilities.

***3.5.2 Relations With Time 3***: The scientific reasoning score defined above at Time 3 was correlated with the scientific reasoning score at Time 2, *r*(157) = 0.32, *p* < 0.001 and Time 1, *r*(145) = 0.23, *p* = 0.004. In addition to the relation with theory of mind performance at Time 3, scientific reasoning at Time 3 was also correlated with theory of mind scores at Time 1, *r*(151) = 0.23, *p* = 0.004, and Time 2, *r*(157) = 0.16, *p* = 0.04, as well as executive function scores at both Time 1, *r*(156) = 0.22, *p* = 0.005, and Time 2, *r*(157) = 0.26, *p* = 0.001. Finally, scientific reasoning at Time 3 was correlated with the PPVT score at Time 3, *r*(153) = 0.29, *p* < 0.001 and SETK score at Time 1, *r*(150) = 0.39, *p* < 0.001.

We conducted a regression with performance on the scientific reasoning score at Time 3 as the dependent variable; performance on the scientific reasoning measures at Time 1 and 2, theory of mind measures at each time point, executive function measures at Times 1 and 2, as well as the SETK and PPVT scores were the independent variables. The overall model was significant, *R* ^2^ = 0.33, *F*(9, 121) = 6.59, *p* < 0.001. Only three individual factors were significant in this model: theory of mind at Time 3, *β* = 0.20, *t* = 2.34, *p = *0.02, executive function at Time 2, *β* = 0.21, *t* = 2.55, *p *= 0.01 and the SETK score at Time 1, *β* = 0.27, *t* = 3.09, *p *= 0.002, although performance on the scientific reasoning measure at Time 1 was marginally significant, *β* = 0.15, *t* = 1.94, *p =* 0.054.^4^

**FIGURE 5:**
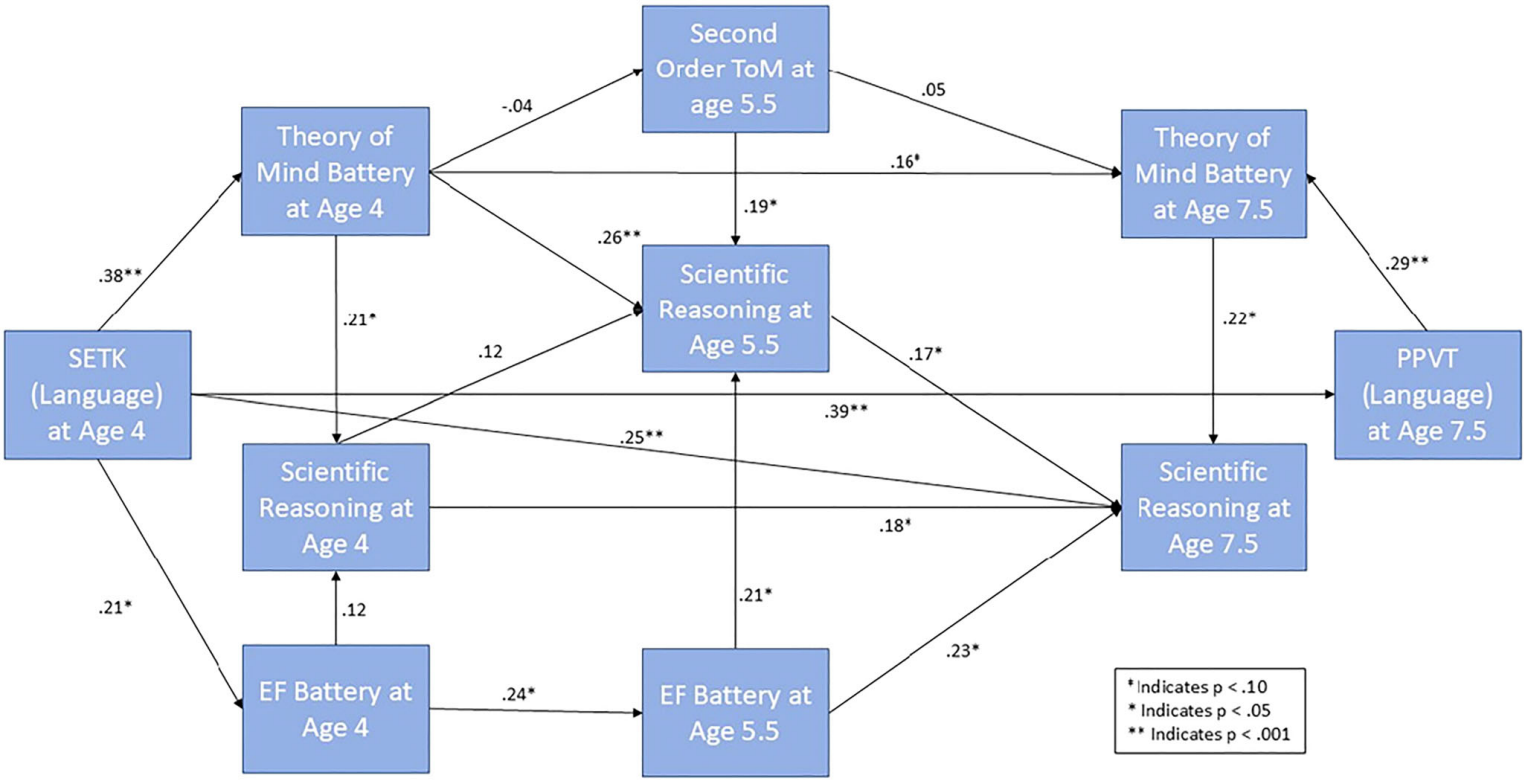
Best fitting path model generated for children's performance on theory of mind, executive function, and scientific reasoning measures (see text for details).

We next considered a path model to identify direct and indirect links among these variables. We built this model projecting paths from earlier times to later times both within domains we investigated (e.g., SETK scores predicted PPVT scores^5^), and between domains based on the findings from our regression analysis; paths were included based on significant results from the regression analyses. For example, PPVT scores were posited to predict theory of mind scores at Time 3, but not scientific reasoning at that time given the regression analysis described above. SETK scores in contrast, were significant in the model predicting scientific reasoning at Time 3, so they were included in the model. This model is shown in Figure 5.

This model presented a good fit to the data, RMSEA = 0.07, *p_close_* = 0.19, CFI = 0.90, TFI = 0.83.^6^ At each time point, there is a significant path from children's theory of mind abilities to their scientific reasoning. Moreover, early theory of mind influences scientific reasoning at age 7.5 both through theory of mind development and through pathways through scientific reasoning abilities at Age 4 and 5.5. Scientific reasoning at Age 4 and Age 5.5 also have significant paths to scientific reasoning at Age 7.5, suggesting that there is some continuity in children's capacities. Finally, the path from executive function at Age 5.5 to scientific reasoning at Age 7.5, suggests that changes in children's executive function between Ages 4 and 5.5 might be critical to their more advanced scientific reasoning; that executive function at Age 7.5 is not a significant predictor of scientific reasoning at 7.5^7^ indicates that once children acquire the more advanced capacity at 5.5, their ability to engage in scientific reasoning improves in complexity, but does not fundamentally change.

The path analysis allows us to make comparisons with several alternative models. The first is a fully concurrent and cross‐lagged model, in which theory of mind and executive function related to scientific reasoning at each time point, and at each subsequent time point either directly or both directly and indirectly. Neither of these were as good a fit to the data as the model shown in Figure 5 (based on BIC, which was 3924.31 and 3939.84 respectively, compared to 3730.21 for the model in Figure 5). Similarly, we considered a model with only concurrent relations with theory of mind and executive function related to scientific reasoning (with only longitudinal relations within either only direct or direct and indirect relations in each category). Neither of these models were better fits (BIC = 3920.13 and 3926.23, respectively), and both had RMSEA values > 0.09, making them inappropriate to consider further.

## 4 Discussion

Children's theory of mind (both RToM and AToM), executive function, and scientific reasoning capacities were measured when they were 4‐years‐old, 5.5‐years‐old, and 7.5‐years‐old. We highlight three findings. First, children's scientific reasoning at Age 4 and 5.5 related to their scientific reasoning at Age 7.5. This finding aligns with longitudinal findings by Osterhaus and Koerber (2023), who showed that scientific reasoning competencies at age 6 explained their more advanced scientific reasoning abilities at Age 10. Early competencies in scientific reasoning may lay the foundation for later, more complex skills within the same domain (e.g., Cunha and Heckman 2007).

Second, at Age 4, the battery of RToM measures related to children's scientific reasoning at that age. Similarly, at Age 7.5, the battery of AToM measures related to children's scientific reasoning at that age. At Age 5.5, children's emerging AToM, but not their RToM ability, related to their concurrent scientific reasoning. Longitudinally, RToM at Age 4 related to children's scientific reasoning at Age 5.5, independent of the emergence of AToM at Age 5.5, children's scientific reasoning at Age 4, or executive function at either age. RToM at Age 4, however, did not directly relate to scientific reasoning at Age 7.5; it appeared to be mediated by AToM performance at that age.

Third, executive function showed independent concurrent relations with scientific reasoning at Ages 4 and 5.5, but critically, a longitudinal relation from Age 5.5 to scientific reasoning at Age 7.5. This longitudinal relation was independent of children's AToM development and earlier scientific reasoning ability.

To interpret these results, at 4‐years‐old, the measure of scientific reasoning required children to identify confounded evidence and select actions to test a causal hypothesis. Following Perner (1991), (2025), and Doherty and Perner (2020), the representational capacities indicated by RToM supports children's ability to represent and coordinate alternative perspectives. Understanding whether data are confounded requires taking the perspective that the outcome could have alternative causal pathways instead of only considering one causal relation.

The scientific reasoning measures administered when children were 5.5‐years‐old reproduced the measure at Age 4 and added a measure in which children explicitly designed interventions to test hypotheses. The experimental design capacity measured in the Ramps task (and the scientific reasoning tasks administered when children are 7.5) requires not only recognizing that data can be interpreted in multiple ways (i.e., what we suggest RToM allows the reasoner), but also what actions would produce such data. This latter capacity emerges as a function of RToM, but as RToM is also the building block for children's AToM, once AToM is more firmly in place at Age 7.5, it mediates any earlier‐developing relation. Thus, between Ages 4 and 7.5, the representational capacity inherent in the development from RToM to AToM indicates the progressive integration of increasingly complex mental‐state representations in children's scientific reasoning capacities.

This interpretation explains why there are no longitudinal relations between scientific reasoning at Ages 4 and 5.5. Children's scientific reasoning capacity is not changing during this time. Their RToM capacity at Age 4 allows them to represent multiple hypotheses, but not construct the interventions necessary to discern between them. This emerges at Age 5.5 as their RToM improves to allow AToM to emerge. Theory of mind development—particularly the development of the ability to represent beliefs—acts as a developmental engine driving the emergence of this more complex scientific reasoning capacity: explicit experimental design requires reflecting on the different hypotheses with alternate possible causes.

Such an interpretation is consistent with findings in domains like reading and math, in which early foundational skills scaffold later abilities, such as letter‐sound recognition supporting vocabulary growth or counting principles facilitating arithmetic. Such skill‐building relies on metacognitive reflection, which is driven by children's developing theory of mind. For example, Kloo et al. (2022) found that numerical knowledge at Age 4 predicted later mathematical abilities at Age 8. Theory of mind capacities at Age 4 also uniquely contributed to the explanation of children's later mathematical abilities. Even older children show benefits of early theory of mind, which potentially relates to prosocial reasoning and the absence of behavioral problems in high school (Weimer et al. 2017). The present findings make a similar conclusion for scientific reasoning: The development of scientific reasoning may rely on the integration of theory of mind with earlier scientific reasoning capacities, rather than solely building upon them.

The interpretation explains two additional facets of the data. First, at Age 4, there was no relation between the ICE and *CVS* measures in the scientific reasoning task. This was similar to the findings of Moeller et al. (2022), who used a wider age range. At Age 5.5, however, performance on these two measures was significantly correlated. This suggests that what is developing is an integration between recognizing data could be confounded and the ability to select interventions designed to disambiguate potential causal models. Second, executive function at Age 5.5 had a significant relation to scientific reasoning, both at this point in time and longitudinally at Age 7.5. This suggests that between the ages of 4 and 7.5, children undergo a transition in their scientific reasoning capacities from simply representing alternate perspectives to acting on those representations in order to resolve ambiguity. Children's planning, working memory, and inhibition skills—the capacities measured by the executive function battery—all facilitate this cognitive processing, which allows children to engage in more complex inferences.

### 4.1 Limitations and Future Directions

We want to highlight three limitations of the present study. First, at each age, we used different measures to assess theory of mind, executive function, and scientific reasoning. A challenge, however, in longitudinal investigations is to present children with age‐appropriate tasks. For example, most 7‐year‐olds perform at ceiling on RToM tasks, which would make re‐administering such measures at this age inappropriate. Using different measures longitudinally did allow us to consider the generality of each measure related to its broader conceptual construct. That is, instead of looking just at longitudinal relations between the Lego task at Age 4 and the Ramps task at Age 5.5, we could also look at the broader relation with scientific reasoning as measured by other tasks.

A second concern is that the scientific reasoning measures used when children were 4‐ and 5.5‐years‐old are less contextualized (i.e., explicitly about scientific content) than the Science K measure used at Age 7.5. The concern with using less contextualized tasks, such as those involving novel causal relations, is that the measure might not generalize to a more contextualized setting, such as a science classroom. That there were specific relations between these measures at earlier ages and the more contextualized measure at Age 7.5 suggests that this concern is unwarranted. Weisberg and Sobel (2022) showed that performance by a set of 3rd graders in the United States (8.5‐year‐olds) on scientific reasoning measures using a similar paradigm to the measure used here with 4‐ and 5;5‐year‐olds, related to their performance in 4th grade on state‐mandated standardized tests of scientific reasoning and content knowledge. This suggests untapped and important relations between the cognitive capacities being tested in these less contextualized measures and the goals of science education.

Third, there is some research suggesting that theory of mind measures, generally construed, do not converge on measuring the same conceptual capacity (e.g., Schaafsma et al. 2015; Warnell and Redclay 2019). Theory of mind is a broad construct that relates to a variety of developing social and cognitive capacities. Here, we treat both RToM and AToM as singular capacities. On the one hand, this method is justifiable given that our factor analyses of these constructed each reduced to single components. On the other hand, we acknowledge that it is possible that other theory of mind measures that could be administered at this age would measure other facets of social cognitive development. For example, Sobel (2023) showed that performance on representational change and false belief questions (i.e., self vs. other RToM measures) have different developmental trajectories, suggesting that these questions on the same unexpected contents task measure different facets of cognition. Future research should tease apart exactly what facets of theory of mind relate to scientific reasoning beyond the measures presented to children here.

## 5 Conclusions

We suggest that as children's RToM emerges, it allows them to represent multiple hypotheses (beliefs about causal structure). Such capacities are part of children's scientific reasoning, and explain the relations observed at Age 4, as well as their development between Ages 4 and 5.5. At Age 5.5, children's AToM begins to emerge, which allows children to not only represent different potential causes of an effect, but also start to reason about what is necessary to disambiguate potential confounds. Between 5.5 and 7.5‐years‐old, there is more continuity in the development of scientific reasoning; performance on executive function measures at 5.5 years related strongly to children's scientific reasoning, and had a cascading effect to Age 7.5. This suggests that children's scientific reasoning capacity does not fundamentally change; children become capable of processing more complex amounts of information. Along with the emergence of AToM, as children's cognitive capacities improve, the complexity of their scientific reasoning follows suit. Theory of mind—and AToM in particular—is potentially a necessary component to the development of scientific reasoning, but not a sufficient one. Other cognitive capacities, which are potentially represented by performance on executive function measures also contribute to children's developing scientific reasoning. This latter finding potentially explains why the context in which a scientific reasoning problem is presented to children might relate to its difficulty. For example, Weisberg et al. (2020) showed 5‐year‐olds could solve problems of interpreting scientific data with few working memory demands, whereas 10‐year‐olds struggled to solve the same problems when the working memory demands increased.

We would speculate that children's theory of mind might play another role in the development of scientific reasoning beyond what we have suggested so far. Theory of mind potentially affords children more advanced social‐collaborative learning. For example, AToM relates with various social factors, such as social competence, social skills, friendship formation, and peer relations (e.g., Lecce et al. 2019; Miller et al. 2018; Weimer et al. 2021) as well as academic achievement (Lecce and Devine 2022). Many scientific activities occur in group settings where children collaborate with peers or teachers to construct knowledge. For example, Schulz et al. (2007) demonstrated that preschoolers were more likely to generate unconfounded evidence for hypothetical causal relations when they were allowed to intervene with a partner than when tested on their own. Theory of mind skill may enhance children's ability to navigate these collaborative environments effectively (Devine et al. 2021; Lecce et al. 2024). Children with better theory of mind skills (both RToM and AToM) may also be more likely to engage in transactive dialogues—peer interactions that challenge ideas and foster cognitive conflict—leading to deeper learning (e.g., Azmitia and Montgomery 1993; Hogan 1999). In group experiments or classroom discussions, theory of mind helps children integrate diverse perspectives and negotiate roles, ultimately enhancing scientific reasoning outcomes.

That said, our findings not only show that AToM measures relate to children's scientific reasoning at an earlier age (5.5) than previously investigated, but also that starting at Age 4, there is a foundational role for the development of RToM abilities on early scientific reasoning. Our focus has been on the ways that theory of mind indicates broader cognitive capacities related to scientific reasoning. We also suggest that social cognition generally construed supports how children might learn from others, and particularly information that cannot be learned from observation and interaction with the world (following Harris and Koenig 2006). Early theory of mind competence, therefore, might facilitate children's learning from others as well as themselves.

## Author Contributions

**April Moeller Bachhuber**: investigation, writing – review and editing, data curation. **Özgün Köksal**: conceptualization, investigation, methodology, writing – review and editing, project administration, visualization, validation, data curation. **Christopher Osterhaus**: conceptualization, methodology, writing – review and editing, investigation. **David M. Sobel**: writing – original draft, conceptualization, methodology, writing – review and editing, formal analysis. **Beate Sodian**: conceptualization, investigation, funding acquisition, writing – review and editing, project administration, supervision, resources, methodology.

## Funding

This research was supported by grant number SO213/34‐1,2 from the German Research Council (Deutsche Forschungsgemeinschaft) to **Beate Sodian**. **David M. Sobel** was supported by grant 2300459 from the National Science Foundation of the United States.

Open access funding enabled and organized by Projekt DEAL.

## Conflicts of Interest

The authors declare no conflicts of interest.

## Supporting information




**Supporting Information**: desc70270‐supp‐0001‐SuppMat.docx

## Data Availability

The data that support the findings of this study are available on request from the corresponding author. The data are not publicly available due to privacy or ethical restrictions.

## Endnotes

1 These are not the only facets of mental state understanding thought to be related to children's scientific reasoning. For example, children's knowledge of the nature of science as a process of learning and belief change as opposed to a set of static facts (e.g., Lederman et al. 2002) relates to their ability to engage in scientific reasoning (Osterhaus and Koerber 2023; Weisberg and Sobel 2022). Children's explicit capacity to revise their beliefs (e.g., Kimura and Gopnik 2019; Macris and Sobel 2017; Young et al. 2012) potentially relates to scientific reasoning, although to our knowledge, this direct link has not been investigated. For the purposes of this paper, however, we will focus on the link between children's developing theory of mind and their scientific reasoning, not their broader developing metacognitive capacities.

2 Osterhaus et al. (2017) also showed that children's AToM related to their understanding of the “nature of science,” another aspect of children's developing metacognition thought to relate to their scientific reasoning (see Footnote 1)

3 There is a literature suggesting that RToM might be present in much younger children in a more implicit manner (e.g., Onishi and Baillargeon 2005). There are multiple interpretations of these results (e.g., Perner and Ruffman 2005). For the purposes of this manuscript, we focus on *explicit* RToM development, in which children can verbalize false belief (or other mental) states.

4 Following the analysis about the AToM measure at Time 2, we also ran this analysis substituting only that second‐order false belief measure instead of all of the theory of mind measures at Time 2. This substitution did not change the nature of the model, and this measure at Time 2 was not significant.

5 There was one exception: Executive function scores at Time 3 are not included in this model, because they did not relate to scientific reasoning scores at this time point. Including these in the model lowered the overall fit of the model (although not to a point where the data were uninterpretable). However, adding this variable does not change any significance levels reported in the model shown, and there is no relation between executive function scores and scientific reasoning at Time 3 in that model.

6 A better fit of the model is obtained if all non‐significant paths between theory of mind at each time point are removed.

7 The absence of EF at 7.5 in the path analysis is based on the lack of significant relations in the regression analysis reported above. When EF at 7.5 is added to this model, the model's overall fit to the data is worse than what is reported.
