# Supplementary material for: Longitudinal Relations Between Representational and Advanced Theory of Mind and Scientific Reasoning From Ages 4 to 7
Source: Dev Sci. 2026 Aug 25;29(5):e70270. doi: 10.1111/desc.70270 (PMC13505288; doi:10.1111/desc.70270)
Supplement: Supplementary file 1 [file DESC-29-e70270-s001.docx]

Longitudinal relations between theory of mind and scientific reasoning from ages 4 to 7

Supplemental Materials Section

**Further Information about the Sample**

Attrition between the time points was within normal expectations for longitudinal research (11% attrition between T1 and T2; 12% attrition between T2 and T3). The main reasons for attrition were lack of time or scheduling difficulties, lack of interest or motivation from the child or family, difficulties in reaching participants, relocation, and personal or health-related issues.

Maternal and paternal education levels were measured at T1. Twenty-two mothers (11%) held a vocational training degree, 25 (12.3%) held a high-school degree, 151 (74.4%) held a university degree, and 3 (1.5%) held an advanced degree. Twenty-three fathers (11%) held a vocational training degree, 28 (14%) held a high-school degree, 148 (73%) held a university degree, and 2 (1%) held an advanced degree. Two families did not answer these questions.

**Detailed Procedures Across the Time Points**

**Theory of Mind Procedures at T1**. The *knowledge access task* assessed children’s ability to understand that others may have access to different information than they do. Children were shown a toy dog figure inside a box and were subsequently questioned about whether a person who lacked visual access to the box would know what was inside of the box. The *content false belief task* (unexpected contents) assesses children’s understanding that others can hold false beliefs about the contents of an object. Children were shown a candy box (Smarties) containing an unexpected toy pig and asked to predict what another child who had not looked into the box would think the box contained. The *location false belief task* (unexpected transfer) assesses children’s ability to understand that others can hold false beliefs about the location of an object. Children were told about a character who holds a belief that his mittens are in a wardrobe, despite them actually being in his backpack, and subsequently, the children were asked to predict where the character would search for his mittens. For each of these tasks, children were scored if they responded correctly or incorrectly.

**Executive Function Measures at T1.** Children’s executive function performance was assessed with two measures. First, we used the *Truck Loading* procedure (originally described by Fagot & Gauvain, 1997, but adapted here from Kloo & Sodian, 2017) to assess children’s planning. Children were presented with a toy mail truck and colored party invitations to deliver to similarly colored houses on a paper playboard. Children had to load the invitations on the truck in reverse order to distribute them in an efficient way. First, the experimenter demonstrated the game using one invitation and one house: “Look, we put the invitation onto the truck and deliver it to the house. But this is a one-way street, so there is only one direction.” Directional arrows on the street were used as a reminder. Then, there was a warm-up with two houses. Children were instructed, “Now there are two houses that we want to invite to the party. The blue invitation goes to the blue house, and the pink invitation goes to the pink house. And we need to deliver these invitations fast so that everyone will be able to come to the party. The fastest way is to drive around only one time.” Then, the experimenter demonstrated loading the truck in reverse order and explained, “You always have to take the letter off the top of the truck so that the top invitation goes to the first house and the next invitation goes to the next house.” A rule check followed (“So, can I take one from the bottom of the truck?”). After children had answered correctly, children delivered the invitations with the experimenter. Then, the test phase began. The warm-up houses were replaced with two differently colored houses (red and green). For each successive level of difficulty, one new house was added (four levels of difficulty or five houses). Each level consisted of two trials. Children had to pass (i.e., loading the truck in correct order) one of them to continue to the next level. On incorrect trials, corrective feedback was given (‘You always have to take the letter off the top of the truck so that the top invitation goes to the first house and the next invitation goes to the next house. And the truck is allowed to move in only one direction’.) Children received a score indicating the highest level of difficulty achieved (0–4). ICC was .96 for this measure.

Second, we used the *Day/Night Stroop task* (Gerstadt et al., 1994) to assess inhibitory control. Small cards depicting either a yellow moon and stars against a black background or a yellow sun against a white background were used. Children were instructed to say ‘‘day” when shown a card with moon and stars and to say ‘‘night” when shown a card with a sun. Then, up to 12 training trials were conducted to ensure that children understood the task. The experimenter presented the cards one after the other, and children received corrective feedback on their answers. After four consecutive correct trials or after a maximum of 12 training trials, testing began. The test phase consisted of 16 cards but was terminated after 12 cards if the training phase had been terminated after four correct responses in a row (and these responses were used for Cards 13 to 16 from the test phase). No feedback was given in the test phase. Children received a score from 0 to 16 based on the number of correct responses in the test phase. ICC was .98 for this measure.

**Measures of General Cognition at T1.** Children’s linguistic memory, sentence comprehension, and morphological rule formation were assessed with three subtests of *SETK 3-5* (Sprachentwicklungstest für Drei- bis Fünfjährige; Grimm et al., 2015). Raw values from each subtest were transformed into standardized t-values according to the age-specific norms and the average of the three subtests was used as an index for children’s verbal ability. ICCs for the three subtests ranged between .83 and .96 for these measures.

**Theory of Mind Measures at T2.** In addition to the measures given at T1, children also received one AToM measure that tested their understanding of second-order false belief. This measure was the birthday puppy story (following Sullivan et al., 1994), which was depicted with colored drawings. In this story, a mother tries to surprise her son (Peter) by misinforming him that he is getting a big, beautiful toy as a birthday gift. In reality, he is getting a puppy. Peter, however, discovers the real present, the puppy, in the basement, unbeknownst to his mother. Children were then asked two control questions, “Does Peter know that he is getting a little dog for his birthday” (correct answer: yes) and “Does Peter’s mother know that Peter found the dog in the basement?” (correct answer: no). Children were then told that Peter’s grandmother calls the mother, and asks whether Peter knows what the mother got him for his birthday, and were reminded that the mother does not know Peter saw the puppy. The grandmother then asks the mother, “What does Peter think he’s getting for his birthday?” and children were asked a second-order false-belief question: “What will the mother tell the grandmother? A beautiful, big toy or a little dog?” (correct answer: a [beautiful, big] toy).

Children received a score of 1 if they answered both the control questions and the second-order false belief question correctly. Otherwise, they received a score of 0. Kappa was 1.00 indicating perfect interrater reliability.

**Executive Function Measures at T2.** Three Executive Function measures were administered at T2 First, children were given a version of the *Dimensional Change Card Sort task* (DCCS; Zelazo, 2006) to assess cognitive flexibility. Children were presented with cards featuring drawings differing in two dimensions: color and shape. In the initial pre-switch phase, they were asked to sort the cards based on the color dimension, and later, in the post-switch phase, the sorting rule was changed to the shape dimension. Then, children were given a more challenging border phase, in which a new dimension was introduced; children were instructed to sort the cards based on the presence or absence of a border: sorting by color when there was a border on the cards and by shape when there was none. Children exhibited ceiling performance in the pre-and post-switch phases, we used their scores from the 12 border version trials. ICC was .99 on this measure.

Second, children were given a *Simon Says* task (originally used by Strommen, 1973, adapted here from Kloo & Sodian, 2017). Children were instructed to perform an action (e.g., touch your nose) only when the instructions were preceded by “Simon says.”. They were particularly told not to perform the action when the instructions were not preceded by “Simon says” (non-Simon trials). We scored the number of times children correctly inhibited their action on the 10 non-Simon trials. ICC was .90 for this measure.

Finally, children were given the *Backwards Digit Span* subtest of the HAWIVA-III (Fritz-Stratmann et al., 2007). Children were presented with a series of digits and asked to repeat the digits in reverse order. Children were initially given a two-digit sequence. If they responded correctly to two sequences of the same length, the length of the sequence was increased by one, up to the maximum of eight digits. Children received a score based on the total number of sequences they could accurately recall in reverse. ICC was .99 for this measure.

**Theory of Mind Measures at T3.** Advanced theory of mind was considered here with three measures. First, the same birthday puppy task used at Time 2 was re-administered. Second, we used the *football trainer* story (Liddle & Nettles, 2006; Osterhaus & Koerber, 2023), which assessed a third-order belief inference. Children were introduced to a character, Niklas, who wants to join the school’s soccer team. Niklas thinks that the coach will choose his friend David over him. The coach thinks that both David and Niklas are good players, and he is aware of Niklas’ concern. Children were asked to judge whether two statements about the coach’s beliefs were true or false: (1) “The coach thinks that Niklas knows that he wants him on the team.” (The correct answer is false), and (2), “The coach thinks that Niklas does not know that he wants him on the team.” (The correct answer is true). The criteria for correctly demonstrating higher-order false-belief reasoning involved answering both questions accurately, scored as a 1. If children did not respond correctly to both questions, they were given a score of 0.

Third, we assessed children’s understanding of nonliteral speech using an adaption of the *double bluff* story (Happé, 1994). Children were presented with a story about a soldier that was captured by his enemies. When his enemies ask about the location of a weapons arsenal, the soldier uses a double bluff to keep its true location hidden: He tells his enemies the weapon arsenal’s true location as he knows they will disbelieve him. Children’s understanding was assessed through their responses to the following forced-choice question: “Why did the soldier say this? A) The soldier said this because he knows that the enemies will not believe him, or B) the soldier said this because that’s where the weapons are hidden.” Children were scored as responding correctly (1) if they endorsed answer A and were scored as responding incorrectly (0) if they gave any other response. Kappas for all three tasks ranged between .90 and 1.00.

**Executive Function Measures at T3.** Children were given the same Backwards Digit Span task as in T2. In addition, children also were a measure of *verbal fluency*, which evaluated their ability to recall information stored in their semantic memory (Roebers et al., 2012). Children were instructed to name as many different members of a given category as possible within a limited time. Initially, they underwent a 10-second training session where they named as many colors as they could. In the two test trials, children first named animals and then named foods within 60 seconds. The unique number of items each child named in each trial was counted. The total verbal fluency score was calculated by summing the word counts from the two test trials. Approximately 30 percent of the data was coded by a second rater, demonstrating excellent interrater reliability (ICC = .99).

Finally, children were administered the *Fruit Stroop* task to assess their inhibitory control (following Roebers et al., 2012). Children were first shown a page of 25 colored squares, and were asked to name the colors as quickly as possible (baseline trials). Next, they were shown color pictures of fruits with their prototypical colors (e.g., a yellow banana). Children were asked to name the colors of each fruit (congruent trials). Next, they were shown the same fruits in black and white, and children were again asked to name the color of each fruit. Finally, children were shown color photos of the same fruits, but in incorrect colors (i.e., a blue banana) and were again asked to name the typical color of the fruit (incongruent trials). We used the same scoring system used by Roebers et al. (2012): Time for Incongruent Trials – [(Time for Baseline x Time for Black & White Trials) / Time for Baseline + Time for Black & White Trials)]. This resulted in a measure in which a lower score indicated better inhibitory control capabilities. Interrater reliability on this measure was excellent (ICC = .98).

**Measures of General Cognition at T3.** For the assessment of children’s verbal ability at T3, we used a German adaptation of the Peabody Picture Vocabulary Test – Fourth Edition (*PPVT-4*; Lenhard et al., 2015). In this task, the experimenter says a word and children should select a picture out of four that best illustrates the word. To reduce the lengthy testing time of the original version, we created a shortened version, which comprised five age-appropriate sets with increasing difficulty (i.e., every second set, from sets 8 to 16, each including 12 words). The shortened version correlated highly with the original version of the test, which was tested based on a subsample, *n* = 24, *r* = .968, *p* < .001. ICC was .96.

**Dimensional Reduction for Theory of Mind, Executive Function, and Scientific Reasoning Measures**

We first considered relations among the theory of mind measures and among the executive function measures administered at each time point via principal component analyses. For the executive function measures at each time point, and for the theory of mind measures at time points 1 and 3, a single component was isolated. For the theory of mind measures at time point 2, the Bartlett’s test of Sphericity was not significant, *p* = .10. This analysis was skewed by performance on the knowledge access measure being at near ceiling levels (94%). When a PCA was conducted without the knowledge access measure, the Barlett’s test was significant, χ^2^(3) = 9.16, *p* = .03, so only the remaining three measures were used to calculate a theory of mind score at that time point^[1]^. Figure S1 shows the relations among each task and the component for that time point. Of note is that all KMO values for these analyses were between .50 and .70, suggesting that there is some uncertainty as to whether these variables each contribute equally to a common factor. As a result, and to account for missing data and cases in which the scoring systems were not equivalent, each measure was *z*-scored, and those *z*-scores were then weighted based on the amount they loaded onto the single factor generated by the PCA into an average score^[2]^. These weighted z-scores were used in the analyses described below to examine relations between theory of mind and executive function on children’s scientific reasoning.

**Figure S1**. Principal Component Analysis results for Theory of Mind and Executive Function Measures at each time point

At time 1, the CVS Lego measure contained three measures – performance on the ICE trials, in which children had to state whether the evidence they observed was sufficient to draw a causal conclusion (34% correct), and performance on two different CVS trials in which children had to choose an intervention that would allow them to know which of two (35% correct) or three (25% correct) blocks had efficacy. A principal components analysis revealed that performance on these three measures reduced to a single factor (i.e., the eigenvalue of the second component was less than 1), but it is important to note that similar to previous findings using these measures (Moeller et al., 2022), performance on the ICE trials and performance on the two CVS trials combined were not significantly correlated, *r*(184) = .09, *p* = .23. Because of the results of the PCA, we considered the overall results to the CVS Lego task in our analysis, reported in the main text, as opposed to dividing the measure further.

Similar to the results at Time 1, a principal component analysis on the three CVS Lego measures at Time 2 revealed that performance on the three measures reduced to only one component. However, unlike Time 1, at Time 2, performance on the ICE trials (62% correct) and performance on the CVS trials involving 2 (40% correct) and 3 (38% correct) blocks were significantly correlated, *r*(164) = .25, *p* = .001. This suggests that at age 5.5, children might have used their understanding of whether data were confounded to make choices about what kind of data they would want to observe in order to make a causal conclusion, where at age 4, children’s reasoning about such data was not as differentiated.

Finally, we performed a principal components analysis on performance on the CVS Ramps and Science K measures at Time 3. Because we did not want to overweigh the 2-item subscale about conclusive and inconclusive tests, we combined it with the CVS subscale, as it was originally administered. These three scores could be reduced to a single factor, so the scores were z-scored and averaged to create a single factor with the Ramps task.

**Analysis of the Scientific Content Measures at Time 2 and 3.**

At Time 2, children’s scientific content knowledge was significantly correlated with their age, *r*(177) = .15, *p* = .05, their executive function score, *r*(177) = .28, *p* < .001, and their scientific reasoning score, *r*(177) = .20, *p* = .007. To consider these relations independently, we analyzed a regression model with children’s scientific content knowledge as the dependent variable and these three factors as the independent variables. The overall model was significant, *R^2^* = .10, *F*(3, 175) = 6.78, *p* < .001. Age was not a significant predictor in this model, β = .11, *t* = 1.50, *p* = .14. Executive function score, however, was a significant predictor, β = .22, *t* = 2.76, *p* = .006 and scientific reasoning score was marginally related, β = .14, *t* = 1.77, *p* = .08.

Performance on the science content measure at Time 3 significantly correlated to children’s theory of mind score at Time 3, *r*(156) = .21, *p* = .009, their scientific reasoning score, *r*(156) = .32, *p* < .001, and PPVT score, *r*(153) = .43, *p* < .001. There was also a gender difference with girls having higher scores than boys, *r*(156) = .17, *p* = .03. To isolate these relations, we constructed a regression model with performance on the scientific content measure as the dependent variable and children’s gender, PPVT score, theory of mind score, and performance on the scientific reasoning tasks as the independent variable. The overall model was significant, *R^2^* = .25, *F*(4, 148) = 12.38, *p* < .001. Gender was a significant predictor, β = .18, *t* = 2.50, *p* = .01, as was children’s scientific reasoning score, β = .22, *t* = 2.80, *p* = .006, and PPVT score, β = .34, *t* = 4.33, *p* < .001. Theory of mind, however, was not a significant predictor in this model, β = .03, *t* = 0.38, *p* = .70.

When we looked at these findings longitudinally, scientific content scores at Time 2 were also significantly correlated with theory of mind scores at Time 1, *r*(170) = .30, *p* < .001, executive function at Time 1, *r*(176) = .20, *p* = .007 children’s language score on the SETK, *r*(167) = .41, *p* < .001, and were marginally correlated with scientific reasoning at Time 1, *r*(162) = .15, *p* = .052. We constructed a regression with scientific content scores at Time 2 as the dependent variable, and all of these factors as independent variables as well as age and gender. The overall model was significant, *R*^2^ = .22, *F*(8, 142) = 4.89, *p* < .001. The SETK score was a significant predictor, β = .28, *t* = 3.32, *p =* .001, as was gender, with girls showing higher scores than boys, β = .16, *t* = 2.14, *p =* .03.

We constructed a similar regression analysis for children’s scientific content scores at Time 3, looking at their scientific content score at Time 2, as well as factors that related to content knowledge at Time 3 (gender, PPVT, and scientific reasoning score at Time 3) as well as factors that potentially showed indirect relations to that performance at Time 3 (e.g., SETK score, theory of mind scores at time 1 and executive function scores at Times 1 and 2). The overall model was significant, *R*^2^ = .34, *F*(8, 132) = 9.03, *p* < .001. In this model, there was unique effects of scientific content at Time 2 predicting scientific content knowledge at Time 3, β = .26, *t* = 3.08, *p =* .002, but also of gender, with girls outperforming boys, β = .18, *t* = 2.52, *p =* .01, and PPVT score, β = .18, *t* = 2.27 *p =* .03, and a marginal effect of theory of mind at Time 1, β = .15, *t* = 1.92, *p =* .06. The Scientific Reasoning score at Time 3 was not significantly related to scientific content knowledge, β = .13, *t* = 1.56, *p =* .12.

Overall, these data suggest that children’s general cognition related to their scientific content scores, but little evidence that scientific reasoning, theory of mind or executive function abilities related to scientific content knowledge.

1. As will become relevant below, the PCA revealed only one component with an Eigenvalue over 1.00. However, the Eigenvalue of the second component was 0.99. When the analysis is forced to extract two components, one component loads strongly on the two first-order theory of mind measures and the second component loads strongly on only the second order theory of mind measure.
2. Because the Fruit Stroop measure used in T3 was inverted (lower scores are better), the scores were inverted for this calculation.
